# Store-Operated Calcium Entry: Shaping the Transcriptional and Epigenetic Landscape in Pancreatic Cancer

**DOI:** 10.3390/cells10050966

**Published:** 2021-04-21

**Authors:** Ana P. Kutschat, Steven A. Johnsen, Feda H. Hamdan

**Affiliations:** 1 Clinic for General, Visceral and Pediatric Surgery, University Medical Center Göttingen, 37075 Göttingen, Germany; ana.kutschat@zentr.uni-goettingen.de; 2 Gene Regulatory Mechanisms and Molecular Epigenetics Lab, Division of Gastroenterology and Hepatology, Mayo Clinic, Rochester, MN 55905, USA; Johnsen.Steven@mayo.edu

**Keywords:** calcium, PDAC, transcription, epigenetics, ER stress, NFAT, SOCE

## Abstract

Pancreatic ductal adenocarcinoma (PDAC) displays a particularly poor prognosis and low survival rate, mainly due to late diagnosis and high incidence of chemotherapy resistance. Genomic aberrations, together with changes in the epigenomic profile, elicit a shift in cellular signaling response and a transcriptional reprograming in pancreatic tumors. This endows them with malignant attributes that enable them to not only overcome chemotherapeutic challenges, but to also attain diverse oncogenic properties. In fact, certain genetic amplifications elicit a rewiring of calcium signaling, which can confer ER stress resistance to tumors while also aberrantly activating known drivers of oncogenic programs such as NFAT. While calcium is a well-known second messenger, the transcriptional programs driven by aberrant calcium signaling remain largely undescribed in pancreatic cancer. In this review, we focus on calcium-dependent signaling and its role in epigenetic programs and transcriptional regulation. We also briefly discuss genetic aberration events, exemplifying how genetic alterations can rewire cellular signaling cascades, including calcium-dependent ones.

## 1. Introduction

Chemoresistance is a main contributor to the low survival rates faced by pancreatic ductal adenocarcinoma (PDAC) patients [1]. Marked by high genomic instability, pancreatic tumors readily adapt to selective pressures, showing high epigenomic and transcriptomic plasticity [2,3,4]. In fact, genomic amplifications of drug-metabolizing enzymes have been observed in pancreatic cancer as well as in other types of cancer as being the main drivers of acquired chemoresistance [5,6]. Interestingly, several other genes are commonly co-amplified during these genomic aberration events, possibly conferring tumors with additional oncogenic properties. For example, upon gemcitabine resistance acquisition in pancreatic cancer as well as in non-small-cell lung carcinoma, an amplification of one of the main targets of gemcitabine, ribonucleotide reductase catalytic subunit M1 (*RRM1*), was observed. This amplification of *RRM1* was accompanied by a gain in copy number and overexpression of other genes, such as stromal interaction molecule 1 (*STIM1*) [5,7]. Interestingly, the co-amplification of *STIM1* induced a shift in calcium signaling, leading to aberrant de-regulation of various transcriptional programs [5]. In fact, calcium signaling has been described to drive key oncogenic processes such as proliferation, migration, invasion and angiogenesis [8,9], but the effects of its de-regulation in PDAC remain largely unknown. Thus, it is plausible that several other genetic aberrations, which would be considered as passenger effects, actually endow the tumor with additional properties. It is therefore of utmost importance to understand and characterize the underlying consequences that various genomic aberrations have in shaping cellular signaling response and transcriptional programs to leverage these dependencies as novel therapeutic targets. Here, we review how selective pressures imposed on pancreatic tumors promote genomic aberrations, which in turn divert the cellular signaling response to stimuli, eliciting transcriptional reprogramming with a primary emphasis on calcium signaling in pancreatic cancer.

## 2. Genetic Amplifications in Pancreatic Cancer

Tumor cells commonly display genomic aberrations such as the amplification or deletion of entire segments of their chromosomes. Such genomic rearrangements are mainly due to failures to conventionally resolve double stranded breaks, while also being a consequence of major catastrophic events [10,11,12]. Thus, copy number variations take place randomly and may be selected for as they provide tumors with additional characteristics. In fact, the development of pancreatic cancer is marked by several genetic aberrations such as *KRAS* mutation and amplification, cyclin-dependent kinase inhibitor 2A (*CDKN2A*) silencing or loss of heterozygosity as well as mutation or loss of tumor protein 53 (*TP53*) and deletion of SMAD family member 4 (*SMAD4*) [13,14,15]. This suggests that many early pro-tumorigenic events take place in the form of genomic aberrations. They shape the cellular transcriptome, while also affecting several processes such as DNA damage response, cell cycle arrest and cellular signaling response.

In addition to its crucial role in the development of cancer, genomic amplifications may affect tumor response to chemotherapy or take place after chemotherapeutic treatment. This may be due to the high genomic instability and plasticity associated with cancer cells. Several studies have described a correlation between the genetic background of a tumor and its responsiveness to chemotherapy. For example, PDAC response to platinum-based agents has been associated with genomic instability and mutation frequency of DNA damage responsive genes, such as *BRCA1* and *BRCA2* [16,17]. Furthermore, major *KRAS* genomic imbalances have been correlated with higher chemotherapy resistance, worse prognosis and more aggressive metastatic subtypes [4]. Key mitogen-activated protein kinase (MAPK) signaling genes, such as epidermal growth factor receptor (*EGFR*), Erb-b2 receptor tyrosine kinase 2 (*ERBB2*) or AKT serine/threonine kinase 1 (*AKT1*), are also commonly amplified in PDAC. Tumors with such alterations displayed higher sensitivity to receptor tyrosine kinase inhibitors, suggesting that these tumors show a greater dependence on MAPK signaling [17,18]. This suggests that the genetic background of cells may highly influence their sensitivity to chemotherapy and shape their cellular dependencies. It further implies that tumors are extremely plastic, favoring certain genetic mutations and/or undergoing major genomic rearrangements as a consequence of selective pressure.

Co-amplifications may reinforce each other or confer independent properties to tumors. Interestingly, some genomic aberration events have been reported to present a high co-occurrence rate, while others have been shown to be mutually exclusive, revealing the importance of the selection applied on tumors. Mutual exclusivity can be a result of genomic aberration redundancy or synthetic lethality, where cells cannot handle both alterations at the same time. On the other hand, co-occurrence of genomic aberrations suggests that the alterations complement each other [19,20]. Thus, characterizing gene amplifications is not only valuable to dissect cancer development, but also to better understand how tumors may adapt to stresses, such as chemotherapeutic agents. Even though specific gene amplifications are regarded as drivers of a certain cancer type or chemotherapeutic resistance, co-amplified genes may also contribute to tumorigenesis. Therefore, considering the genetic background is crucial, while dissecting the interplay between genomic aberrations may widen our understanding of cancer and cancer cell plasticity.

One such example is the amplification of *ERBB2* in breast cancer. The amplified region extends over 100 KB on chr17 and encompasses not only *ERBB2*, but also several other genes including *TCAP*, *PNMT*, *PGAP3*, *MIEN1* and *GRB7* [21,22]. Later studies revealed that the co-amplification of *MIEN1* facilitates increased migratory potential in breast cancer in a HER2-independent manner. In this case, MIEN1 was shown to promote migration by triggering downstream Syk-dependent signaling and interacting with Annexin A2 [23,24]. On the other hand, the overexpression of *GRB7* further augments the effects of HER2 overexpression. GRB7 interacts with HER2 directly via its SH2-domain, and activates Ras triggering the initiation of the MAPK signaling cascade upon EGF signaling [25,26]. This reinforces the dependency of *HER2* amplified tumors on MAPK signaling, making them especially vulnerable to HER2 and MAPK inhibitors [27]. Thus, the overexpression of co-amplified genes may synergistically promote tumorigenesis or endow cells with different oncogenic properties. Furthermore, by co-amplifying several components of a signaling cascade, cancer cells rewire their response to stimuli and present a shift in their dependencies.

Another example of such an occurrence is the co-amplification of *WEE1* and *ILK* on chromosome 11 upon acquired gemcitabine resistance [5]. The overexpression of WEE1 may augment gemcitabine resistance, as gemcitabine targets DNA polymerase triggering replication stress, while WEE1 is known to protect cells from DNA damage by inducing cell cycle arrest [28,29]. This way, the upregulation of WEE1 could play a cytoprotective role upon gemcitabine treatment. In fact, WEE1 inhibition in combination with gemcitabine and radiation therapy has shown promising results in pancreatic cancer patients [30,31]. On the other hand, ILK has been shown to promote cell proliferation and migration by activating the Akt/mTOR and glycogen synthase kinase 3β (GSK-3β) pathways, while also fostering the expression of Snail family transcriptional repressor 1 (SNAI1) [32,33]. Furthermore, ILK inhibition in pancreatic cancer has been proven to prevent tumor growth and to synergize with acute gemcitabine treatment [34]. Thus, while the overexpression of WEE1 and ILK may both contribute to gemcitabine resistance, they likely elicit different dependencies on cell cycle and Akt/mTOR signaling, respectively. Still, the exact cellular properties conferred by *WEE1* and *ILK* amplification upon gemcitabine resistance in PDAC have not been addressed.

Similarly, other genes amplified upon gemcitabine resistance in pancreatic cancer are *RRM1* and *STIM1* [5]. The amplification and overexpression of *RRM1* was shown to drive gemcitabine resistance, while the upregulation of *STIM1* elicits a shift in calcium signaling, promoting ER stress resistance and aberrant transcriptional activation [5]. This deregulation elicits a crucial shift in pancreatic cancer, as calcium is a major second messenger of several intracellular signaling cascades, influencing various cellular processes, such as transcription, metabolism, stress response [8,9,35]. Consistently, the de-regulation of calcium signaling and homeostasis is tightly associated with pancreatitis, which is a major risk factor for pancreatic cancer [36,37,38,39,40], and has been suggested to take place early in PDAC development [41]. In fact, several genes associated with calcium signaling have been shown to be differentially methylated upon pancreatic cancer development [41]. Thus, there is a need to better elucidate the role of calcium signaling deregulation in pancreatic cancer.

## 3. The Role of Calcium in the Cell

The importance of calcium in mediating cellular responses to environmental stimuli was first reported in 1883, where electrolytes were described to be essential for muscle contraction [42]. Since then, great advances in calcium signaling have been made and its importance in a variety of cellular processes recognized. The intensity of calcium uptake by the cell and its organelles, together with the amount of calcium released by cellular stores, determines the kinetics and intensity of the response to a stimulus. Furthermore, changes in calcium levels in different cellular compartments can have drastic consequences. Increases in cytoplasmic calcium levels can trigger the activation of several calmodulin-dependent proteins culminating in the activation of a diverse range of transcription factors [35,43,44,45].

### 3.1. Calcium-Dependent Signaling

**Figure 1:**
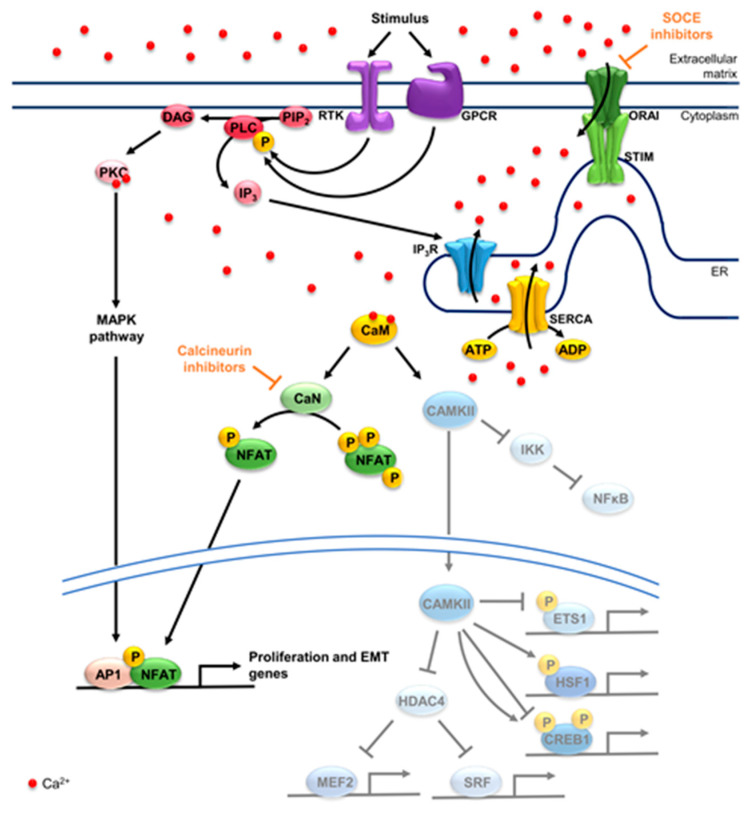
Calcium-dependent signaling and transcription factors. Upon a stimulus, GPCRs or RTKs are activated, phosphorylating PLC and promoting the breakdown of PIP2 to DAG and IP3. The latter promotes the opening of the channel IP3R, raising cytosolic calcium levels. STIM and ORAI further promote the influx of calcium into the cytoplasm through SOCE. DAG and calcium activate PKC and consequently the MAPK pathway, fostering AP1 activity. SERCA pumps calcium from the cytoplasm into the ER negatively regulating PKC. CaM binds calcium, activating CaN and CAMKII. CaN dephosphorylates NFATs, promoting its translocation into the nucleus, where it can dimerize with AP1, among other factors, to activate target genes. In the cytosol, CAMKII activates NFκB by phosphorylating IKK2. CAMKII further activates HSF1, while repressing ETS1. CREB1 can be phosphorylated by CAMKII at two sites, one repressive (Ser142) and one activating (Ser133). CAMKII inhibits HDAC4, promoting the activation of MEF2 and SRF. SOCE inhibitors can be employed to target STIM1 or ORAI1 and consequently calcium-dependent programs, while calcineurin inhibitors block NFAT activation. In yellow are calcium pumps, in blue calcium channels and in green the components of SOCE, STIM and ORAI. Concerning signaling components, receptors are in purple and their downstream effectors in pink/red. Factors activated by CaN in green and factors activated and/or repressed by CAMKII in blue. GPCR: G protein-coupled receptor; RTK: receptor tyrosine kinase; PLC: phospholipase C; PIP2: phosphatidylinositol 4,5-bisphosphate; DAG: diacylglycerol; IP3: inositol 1,4,5-triphosphate; IP3R: IP3 receptor; STIM: stromal interaction molecule; ORAI: calcium release-activated calcium channel; SOCE: store-operated calcium entry; PKC: protein kinase C; MAPK: mitogen-activated protein kinase; AP1: activator protein 1; SERCA: sarco/endoplasmic reticulum calcium-ATPase; ER: endoplasmic reticulum; CaM: calmodulin; CaN: calcineurin; CAMKII: calcium/calmodulin-dependent protein kinase II; NFAT: nuclear factor of activated T cells; NFκB: nuclear factor-kappa B; IKK2: inhibitor of nuclear-factor kappa B kinase subunit beta; HSF1: heat shock factor 1; ETS1: c-ets-1; CREB1: cAMP response element-binding protein 1; HDAC4: histone deacetylase 4; MEF2: myocyte enhancer factor 2; SRF: serum response factor.

In non-excitable cell systems, G protein-coupled receptors (GPCRs) and receptor tyrosine kinases (RTKs) relay external events to the cell often by activating calcium-dependent signaling cascades in the cell. Upon substrate binding, GPCRs and RTKs are activated and lead to phosphorylation of phospholipase C (PLC). Once activated, PLC hydrolyzes phosphatidylinositol 4,5-bisphosphate (PIP_2_) into inositol 1,4,5-triphosphate (IP_3_) and diacylglycerol (DAG) [46,47]. IP_3_ then binds to its receptor (IP_3_R) in the ER, promoting the release of calcium from the ER into the cytoplasm. As cytoplasmic calcium levels increase, protein kinase C (PKC) binds calcium and DAG, thereby, becoming activated. PKC in turn phosphorylates several targets such as components of the MAPK pathway, amplifying the stimulus sensed by GPCRs and RTKs [48,49]. In this way, PKC activity and the overall signaling intensity are directly modulated by cytoplasmic calcium levels. Consequently, several calcium channels, pumps and exchangers modulate PKC activity (Figure 1).

STIM and calcium release-activated calcium channel (ORAI) positively regulate PKC by sensing calcium depletion from the ER. STIMs are ER transmembrane proteins which oligomerize upon ER calcium store depletion, activating ORAI at ER-cytoplasmic membrane junctions [50,51]. ORAI channel opening promotes the increase in cytoplasmic calcium levels in a process called store-operated calcium entry (SOCE) [51]. Alternatively, STIMs can activate canonical transient receptor protein (TRPC) together with ORAI, promoting the store-operated influx of calcium as well as other cations from the extracellular matrix into the cytoplasm [52]. In turn, ER calcium levels are restored by sarco/endoplasmic reticulum calcium-ATPase (SERCA), which hydrolyze ATP in order to transport calcium ions against the concentration gradient back into the ER [53] and thereby negatively regulate PKC activity [49]. Thus, GPCRs and RTKs rely on an increase in cytosolic calcium levels to activate PKC and the MAPK pathway. Consequently, the kinetics of the response are tightly regulated by calcium pumps, channels, and exchangers in all cellular compartments where their activities are closely interconnected.

### 3.2. Calcium-Dependent Transcription Factors

Increased cytoplasmic calcium levels lead to the activation of calmodulin-dependent proteins such as kinases and phosphatases. These, in turn, promote the activation or inactivation of several other transcription factors [54,55] (Figure 1). One of these important kinases is calcium/calmodulin-dependent protein kinase II (CAMKII) which translocates to the nucleus and phosphorylates calcium/calmodulin-dependent protein kinase IV (CAMKIV) upon activation. CAMKII and CAMKIV further phosphorylate and activate several transcription factors in the cytoplasm and the nucleus [56]. In the cytoplasm, CAMKII releases the inflammation-associated transcription factor nuclear factor-kappa B (NFκB) from an inhibitory complex by activating inhibitor of nuclear-factor kappa B kinase subunit beta (IKK2) [57,58,59]. Once in the nucleus, cAMP response element-binding protein (CREB) is activated upon phosphorylation of Ser133 by CAMKIV, while CAMKII can phosphorylate Ser133 or Ser142 of CREB, thereby activating or inhibiting it, respectively [60,61]. CREB hyperactivation has been tightly associated with cancer initiation, development and metastasis [62,63]. Another common target of CAMKII and CAMKIV is the serum response factor (SRF), which is activated upon phosphorylation by both enzymes [64,65], and is best characterized for driving key cell migration genes [66,67]. CAMKII activity is also linked to the cellular stress response, as it phosphorylates and activates the stress responsive transcription factor heat shock factor 1 (HSF1) at Ser230 [68,69]. HSF1 has been shown to not only enable tumor cells to thrive under stress, but to also foster cell proliferation, invasion and migration, while dampening inflammation and apoptotic programs [70,71]. Protein c-ets-1 (ETS1) is also inhibited upon phosphorylation by CAMKII [72,73]. ETS1 is best known for fostering cell invasion and playing an important role in angiogenesis, while also promoting epithelial-to-mesenchymal transition (EMT) in several cancers including PDAC [74]. Interestingly, CAMKII and CAMKIV can also phosphorylate histone deacetylase 4 (HDAC4) promoting its nuclear export. HDAC4 not only deacetylates histones but can also inhibit SRF and myocyte enhancer factor 2 (MEF2). This way, CAMKII and CAMKIV indirectly activate SRF, while also promoting the activity of MEF2, whose oncogenic or tumor suppressive roles remain to be further investigated [54,75].

The phosphatase calcineurin (CaN) also becomes active upon SOCE stimulation, dephosphorylating transcription factors which are usually kept inactive in the cytoplasm by phosphorylation. CaN can also be inhibited upon phosphorylation by CAMKII in a negative feedback loop [76,77]. CaN has been suggested to foster MEF2 activity when active, although the exact mechanism is still under investigation [78]. The main substrate of CaN is the nuclear factor of activated T cells (NFAT) family, which is crucial for T cell activation, differentiation and development, while also promoting cancer development [9,79]. Upon dephosphorylation by CaN, the nuclear localization signal of NFAT protein is unmasked, allowing NFATs to translocate into the nucleus and activate target genes [79]. Interestingly, NFATs have been described to dimerize with several members of the activator protein 1 (AP1) family of transcription factors including FOS and JUN, converging in a transcriptional program that is highly dependent on both MAPK and calcium signaling [80,81,82]. This may be of particular relevance to pancreatic tumors that highly depend on both mutant KRAS signaling and heightened SOCE.

In PDAC, NFATs have been extensively characterized as drivers of pancreatic cancer development and growth. In fact, NFATc1 activation, together with aberrant KRAS signaling, has been shown to drive acinar to ductal metaplasia [83]. The promoter of *NFATc1* itself has been shown to be methylated by *Enhancer of Zeste* Homolog 2 (EZH2) and consequently silenced in pancreatic acinar cells. This was reversed upon KRAS activation during PDAC development, leading to the de-repression and concomitant activation of NFATc1 [84]. Importantly, NFATs then drive the expression of the ductal determining factor SRY-box transcription factor 9 (*SOX9*) fostering acinar to ductal metaplasia [83,85]. Furthermore, NFATs and signal transducer and activator of transcription 3 (STAT3) have been shown to be activated upon inflammation promoting inflammation-driven pancreatic cancer development. NFATc1-STAT3 dimers were shown to act on enhancer regions, promoting the transcription of Wnt family members, *EGFR*, and several other genes associated with stemness, migration, invasion and growth [86]. NFATc2 has also been shown to promote the silencing of the tumor suppressor *CDKN2B*, further fostering tumorigenesis [87].

In pancreatic cancer, NFATs have been described to promote cell proliferation and EMT. For example, transforming growth factor β (TGFβ) has been reported to activate NFAT, which in turn displaces SMAD family member 3 (SMAD3) repressor complexes allowing for the upregulation of *MYC* and ultimately promoting tumor growth [88,89]. Interestingly, NFAT binding to the *MYC* promoter region, following serum treatment, also fosters p300-depedent acetylation of nearby histones. This, in turn, facilitates the recruitment of ETS like-1 (ELK1) and allows for the upregulation of *MYC* [90]. Finally, NFATc1 has been shown to drive SRY-box transcription factor 2 (*SOX2*) expression, while also dimerizing with it and promoting metastasis by regulating EMT genes, such as zinc finger E-box-binding homeobox 1 (*ZEB1*) and *SNAI1* [91]. Taken together, NFATs play a determining role in pancreatic cancer development, activating key proliferation as well as EMT genes.

In summary, calcium affects various transcription factor programs that are implicated in different cellular processes. Additionally, calcium was reported to play an important role in stress response, which frequently occurs in tumors.

### 3.3. Calcium and the ER Stress Response

ER stress results from protein chaperones in the ER responding to the accumulation of unfolded proteins triggered by internal as well as external cues. These stimuli encompass, among others, disturbances in the oxidizing environment and calcium levels in the ER as well as overall cellular ATP levels and culminate in the accumulation of unfolded proteins. Proteins being folded in the ER contain several disulfide bonds, requiring an oxidizing environment. Thus, failure to recycle protein disulfide isomerases upon oxidative stress leads to an accumulation of misfolded proteins which lack disulfide bonds. Furthermore, protein chaperones need ATP to bind and release proteins, coupling protein folding to overall energy levels in the cell. Calcium is a second messenger, which is stored in the ER and released upon distinct signals, including ER stress. It is buffered and used as a co-factor by protein chaperones, linking ER stress to several cellular processes such as apoptosis, oxidative phosphorylation and transcriptional activation [35,92,93].

In fact, ER stress is commonly characterized by aberrant ER calcium store depletion resulting from increased ER calcium leakage or IP_3_R activity [35]. This drop in ER calcium levels triggers the opening of ORAIs and TRPCs replenishing the ER with calcium and alleviating ER stress in macrophages [94]. Similarly, increased SOCE in STIM1-overexpressing cancer cells renders them resistant to ER stress [5], while decreased STIM1 and SOCE levels promote ER stress in pancreatic β cells [95]. Interestingly, ORAI1 and SOCE levels have been associated with increased ER stress-driven apoptosis in prostate cancer [96]. Thus, calcium signaling in the cytoplasm and the ER are tightly coupled to ER stress in a context-specific manner.

Moreover, the interface between the mitochondria and the ER, namely mitochondria associated membranes (MAMs), allows calcium to easily flow from the ER to the mitochondria, influencing energy production, reactive oxygen species (ROS) formation and cell death initiation. Upon several stimuli, ER calcium is released through the IP_3_R channel and taken up by the mitochondrial calcium uniporter (MCU) [9,43]. High frequency calcium oscillations result in the activation of calcium-dependent enzymes, boosting oxidative phosphorylation and thereby, ATP production. Still, a continuous overflow of calcium in the mitochondria can trigger apoptosis [35]. Thus, calcium flux in MAMs modulates oxidative phosphorylation as it is tightly linked to the maintenance of the oxidizing environment in the ER and to the production of ROS, a known trigger of ER stress [97]. Notably, decreased levels of the ER protein disulfide isomerases, thioredoxin related transmembrane protein 1 and 3 (TMX1 and TMX3), in melanoma cells have been shown to lead to less MAMs, lowered ER–mitochondria calcium flux and increased ROS levels [98]. This underscores the role of calcium and MAMs in redox homeostasis and its coupling to ER protein folding. Furthermore, ER stress mediators have been tightly associated with MAMs and have been suggested to modulate ER–mitochondria communication [35]. Thus, calcium signaling in MAMs fine tunes ER homeostasis, directly affecting ER protein folding and consequently triggering and shaping ER stress response.

In the ER, protein chaperones buffer almost all calcium in the ER, while also using it as a co-factor. One such example is Heat Shock Protein Family A (Hsp70) Member 5 (GRP78), which alone buffers about 25% of ER calcium [99]. Furthermore, the glycoprotein chaperones Calnexin and Calreticulin also bind calcium whereby the structure of the C-terminal domain of Calreticulin is highly dependent on how many calcium ions it binds and overall calcium levels in the ER [100]. Calcium buffering in the ER and in the cell is essential as it controls the amount of calcium which can be released upon a stimulus, fine tuning the cellular response to stimuli [101]. Furthermore, the chaperoning activity of GRP78, Calnexin and Calreticulin depends on calcium and is reduced in response to low calcium levels in the ER. Thus, calcium levels directly influence protein chaperoning activity and ER stress activation [102,103,104].

**Figure 2:**
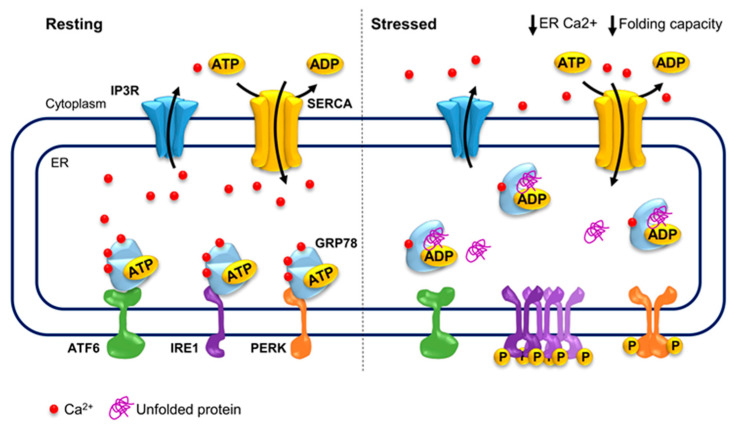
ER stress and its calcium dependency. During resting conditions, GRP78 inhibits the activation of ER stress mediators, ATF6, IRE1 and PERK, which in turn hinder the ATPase activity of GRP78. Furthermore, GRP78 buffers 25% of ER calcium and calcium levels highly modulate the protein folding activity of GRP78 and other protein chaperones. Upon stress, GRP78 dissociates from ER stress mediators, leading to their activation and triggering the ER stress response. GRP78: Heat Shock Protein Family A (Hsp70) Member 5; ER: endoplasmic reticulum; ATF6: Activating Transcription Factor 6; IRE1: inositol requiring enzyme 1; PERK: PKR-like ER kinase.

GRP78 is the main sensor of unfolded proteins and direct regulator of ER stress mediators, linking the cellular energy and ER calcium levels to ER stress. Under resting conditions, GRP78 inhibits ER stress mediators by directly interacting with their luminal domains, which in turn hinder the ATPase activity of GRP78. GRP78 binds unfolded proteins and hydrolyzes ATP to ADP upon stress, trapping the unfolded protein and dissociating from ER stress mediators triggering ER stress. Only after successful folding of the protein and exchanging ADP for ATP is GRP78 able to inhibit ER stress mediators again [105,106]. Thus, GRP78 is the main activator of the stress response, being the major sensor of accumulated unfolded proteins in the ER, and whose activity is directly modulated by cellular ATP as well as ER calcium levels (Figure 2).

### 3.4. ER Stress Responsive Transcription Factors

ER stress mediators are the bridge between ER homeostasis and the stress response, activating the bZIP transcription factors: Activating Transcription Factor 6 (ATF6), X-Box-Binding Protein 1 (XBP1) and ATF4. The ER stress mediators are comprised of three transmembrane ER proteins: ATF6, inositol requiring enzyme 1 (IRE1), and PKR-like ER kinase (PERK). While ATF6 activates UPR-responsive genes including mainly protein chaperone genes, IRE1 leads to the activation of c-Jun N-terminal Kinases (JNK) as well as of XBP1, promoting apoptotic programs as well as the upregulation of protein chaperone, lipid synthesis and ER-associated protein degradation (ERAD) genes [107,108,109]. PERK, in turn, activates the transcription factor Nuclear Factor Erythroid 2-related Factor 2 (NRF2), while triggering ATF4 accumulation [110,111].

#### 3.4.1. ATF6

ATF6 is an ER-transmembrane protein that is vesicle transported to the Golgi apparatus upon ER stress. Site-1 protease (S1P) cleaves off the luminal domain of ATF6 in the ER and site-2 protease (S2P) removes the transmembrane anchor of ATF6. The N-terminus of ATF6 is then released into the cytosol translocating into the nucleus and activating several ER protein chaperone genes such as *GRP78* and protein disulfide isomerase family A member 6 (*PDIA6*) [108,112,113,114]. Thus, ATF6 not only senses ER stress, but also activates stress-responsive genes to promote ER protein folding.

#### 3.4.2. XBP1 and JNK

IRE1 is a kinase and endoribonuclease, which targets several mRNAs and miRNAs in a process called regulated IRE1-dependent decay (RIDD), while also excising an intron from the XBP1 mRNA [115,116]. IRE1 autophosphorylates in trans upon ER stress, leading to its oligomerization and stabilizing its RNase active site. Oligomerized IRE1 binds TNF receptor-associated factor 2 (TRAF2), leading to the activation of JNK, and the promotion of apoptosis by stimulating pro-apoptotic protein Bcl-2-like protein 11 (BIM) and inhibiting anti-apoptotic protein B-cell lymphoma 2 (BCL2) [117,118]. IRE1 further fosters pro-survival pathways by promoting the accumulation of XBP1. IRE1 splices out an intron of the XBP1 mRNA, leading to a shift in the open reading frame, such that the mRNA can be properly translated [119,120,121]. XBP1 then translocates to the nucleus where it activates many pro-survival genes including protein chaperones, ERAD subunits and lipid synthesis protein coding genes. Consequently, XBP1 promotes the proper folding of newly synthesized proteins and the degradation of unfolded proteins while also stimulating the production of phospholipids for ER membrane expansion [109,122]. Thus, IRE1 triggers pro-survival as well as apoptotic transcriptional programs in response to ER stress.

#### 3.4.3. ATF4 and NRF2

The third ER stress mediator is PERK, which senses unfolded proteins in the ER and undergoes oligomerization and autophosphorylation. PERK phosphorylates itself as well as NRF2 and eukaryotic translation initiation factor 2 alpha subunit (eIF2α) [123,124,125,126,127]. Phosphorylation of NRF2 promotes its translocation to the nucleus and activates metabolic enzymes and antioxidant protein coding genes [123,124,128]. The phosphorylation of eIF2α by PERK leads to an inhibition of global cap-dependent translation and a cap-independent translation of ATF4, triggering the transcription of pro-survival and apoptotic genes.

ATF4 has been described to upregulate the expression of several autophagy genes such as microtubule-associated proteins 1A/1B light chain 3B (*MAP1LC3*), autophagy-related 5 (*ATG5*) and sequestosome 1 (*SQSTM1*) [129,130]. This helps catabolize proteins, replenishing the pool of amino acids, while also lowering the amount of unfolded proteins, thereby further alleviating stress [131,132]. Upon prolonged stress, ATF4 upregulates DNA damage-inducible transcript 3 (*DDIT3*), which encodes the transcription factor CHOP. In response to ER stress, ATF4 and CHOP have been shown to promote the transcription of several pro-apoptotic genes such as the Bcl-2 family members Bcl-2-binding component 3 (*PUMA*) and *BIM* [133,134]. Another important target gene of ATF4 and CHOP is tribbles pseudokinase 3 (*TRIB3*), which was shown to repress tumorigenesis and promote apoptosis by inhibiting AKT activation [135,136]. Furthermore, ATF4 and CHOP cooperatively promote the transcription of protein phosphatase 1, regulatory subunit 15A (*GADD34*), whose main substrate is peIF2α and which negatively regulates PERK-dependent ER stress response [126,137]. Thus, PERK activates NRF2 and leads to an antioxidant response and ATF4 accumulation upon ER stress, thereby promoting the transcription of pro-survival and apoptotic genes.

Taken together, ER stress is triggered by several stimuli including changes in ER calcium levels, which impart protein chaperoning activity eliciting an accumulation of unfolded proteins. This, in turn, leads to the activation of different ER stress mediators and a wide range of transcription factors which can then fine-tune the stress response and trigger the transcription of pro-survival genes or, if the cell fails to respond, of apoptotic genes.

### 3.5. ER Stress Response: Essential or Dispensable for the Tumor?

Several studies have pointed at the fact that tumors may take advantage of environmentally induced stress by hijacking the pro-survival branch of the stress response. In fact, the pro-survival ATF4-triggered response has been associated with several tumorigenic processes such as angiogenesis, metastasis, genomic instability, and therapeutic resistance [115,138,139,140,141]. One of the best characterized examples is hypoxia-triggered ER stress response and the subsequent adaptation to oxygen-deprivation. In this case, ATF4 activation has been proposed to be crucial for the transcription of angiogenic genes. Both ATF4 and XBP1 have been reported to bind the promoter region of vascular endothelial growth factor A (*VEGFA*) and ATF4 has been shown to be a driver of *VEGFA* expression upon stress [142,143]. Thus, it is possible that highly hypoxic tumors such as PDAC rather profit from the activation of ATF4.

Following this hypothesis, it is possible that PDAC tumors displaying genetic amplifications, which confer a dampened ER stress response, may be more vulnerable to hypoxia. While genetic amplifications, such as the overexpression of *STIM1*, may protect pancreatic cancer cells from the pro-apoptotic branch of the ER stress response [5], they may potentially also impede them from hijacking the pro-survival branch upon hypoxia and other ER stresses. One could speculate that these tumors would fail to activate ATF4, thus failing to upregulate *VEGFA* and to stabilize HIF1a upon hypoxia.

On the other hand, STIM1 and SOCE have been shown to promote HIF1a expression and stabilization during hepatocarcinogenesis. In this case, upon hypoxia, elevated SOCE leads to the activation of CAMKII and subsequently of the p300 acetyltransferase [144]. p300, in turn, interacts with the transactivation domain of HIF1a, stabilizing and preventing its degradation [145]. Interestingly, in a feedback mechanism, HIF1a has been shown to bind the promoter region of *STIM1* increasing its expression upon hypoxia. Furthermore, several different tumor types have shown a positive correlation between STIM1 and HIF1a levels [144,146]. Thus, it is also plausible that some tumors rely on the activation of SOCE by STIM1 to cope with hypoxia.

For this reason, tumor cells harboring an amplification of *STIM1* may be able to thrive under hypoxic conditions by activating HIF1a through SOCE, circumventing the dampened ER stress response and ATF4-dependent HIF1a activation. In this case, the benefits of hijacking the pro-survival pathway of the ER stress response upon hypoxia may become dispensable for the cell. Instead, tumors may employ SOCE-dependent alternative mechanisms and pathways to cope with oxygen-deprivation. Thus, redundant signaling pathways may aid tumors with very different genetic and epigenetic backgrounds to adapt to the same source of stress. This may apply not only to hypoxia-induced stress, but also to other external stimuli.

## 4. The Benefits and Drawbacks of Targeting Calcium Signaling in Pancreatic Cancer

As described in the sections above, co-amplifications acquired by tumors may divert the cellular signaling response and therefore the transcriptional dependencies of cancer cells. Calcium is an important second messenger involved in relaying diverse signals in the cell, ultimately leading to the activation or inhibition of a vast array of transcription factors. Thus, genomic aberrations involving calcium regulators may result in an altered calcium signaling response and an aberrant activation or repression of transcription. One such example is that of the co-amplification of *STIM1* in pancreatic cancer cells upon the acquisition of gemcitabine resistance, which elicits a shift in calcium signaling and transcription factor dependencies [5]. The consequent increased SOCE and NFAT activity may confer the cells with several additional oncogenic properties, while the dampened response to ER stress protects the cells from apoptosis. Thus, genetic amplifications modulating calcium signaling elicit novel dependencies in tumors, which might be exploited for targeted therapy. In fact, the inhibition of SOCE or NFAT may prevent the activation of pro-tumorigenic processes and transcription factors, while targeting SOCE may also re-establish ER stress sensitivity (Figure 1).

NFAT activation can be targeted by inhibiting calcineurin activity with cyclosporine A (CSA) or FK506 (tacrolimus). These compounds are routinely used in the clinic as immunosuppressants, preventing organ transplantation rejection, and their potential in cancer treatment is still under investigation [147,148]. Studies have shown the benefits of using these FDA-approved drugs in the treatment of different cancer entities. In bladder cancer, both CSA and tacrolimus led to decreased migration and invasion in vitro as well as decreased tumor volume in vivo [149]. In breast cancer, tacrolimus treatment reduced cancer cell proliferation and migration, while inhibiting angiogenesis [150]. Still, the use of these compounds for cancer treatment has to be carefully assessed and treatment schedules wisely planned. Prolonged exposure to CSA or tacrolimus has been associated with increased cancer incidence [151,152]. However, both compounds elicit severe side effects [147,153]. Still, CSA or tacrolimus treatment would solely inhibit potential pro-oncogenic pathways that arise due to aberrant NFAT activation without affecting the aberrant activation or repression of other calcium-dependent transcription factors, while also not influencing other calcium-dependent cellular processes such as the ER stress response.

Thus, SOCE inhibitors could be employed in order to target the potential pro-oncogenic properties elicited by aberrant NFAT activation, while also blocking the aberrant activation or repression of other calcium-dependent transcription factors and sensitizing tumor cells to ER stress. In fact, treatment with the SOCE inhibitor RP4010 in pancreatic cancer led to reduced NFAT translocation and increased expression of eukaryotic translation initiation factor 4E-binding protein 1 (*EIF4EBP1*) [154]. Interestingly, ATF4 and MYC have been shown to upregulate *EIF4EBP1* in order to cope with proteotoxic stress [116,155], suggesting increased activation of ATF4 upon RP4010 treatment. Furthermore, SOCE inhibition in PDAC has been shown to synergize with the standard chemotherapeutic agents, gemcitabine and nab-paclitaxel, in vitro as well as in patient-derived xenografts [154]. Even though no SOCE inhibitor has been approved by the FDA yet, several have been developed and some are being tested in clinical trials for the treatment of acute pancreatitis and pneumonia [156,157,158,159,160,161]. During preclinical studies, the SOCE inhibitors GSK7975A and CM128 have shown very promising results in treating acute pancreatitis while also preventing pancreatic acinar cell injury [162,163]. As clinical trials with CM4620 (also known as Auxora) and other SOCE inhibitors evolve, scientists may better gauge the impact of SOCE inhibition in the body. It is highly possible that SOCE inhibitors lead to the immunosuppression of patients, as SOCE is upstream of NFAT activation, and as the immunosuppressants CSA and tacrolimus are known repressors of NFAT activity. Furthermore, several important SOCE-dependent physiological processes such as the release of insulin in the pancreas, the glomerular hemodynamics in the kidney, the formation of osteoclasts in the bone and the differentiation of myoblasts in skeletal muscle may be transiently impaired during SOCE inhibitor treatment [164]. Still, the occurrence and severity of these potential side effects will largely depend on the concentration and duration of the inhibitor treatment.

Assuming CM4620 and/or other SOCE inhibitors are approved by the FDA and little to mild side effects are detected, SOCE inhibitors may be suitable candidates to treat pancreatic tumors in which STIM1 is overexpressed. Thus, assessing the expression of STIM1 in patient biopsies before and after chemotherapy may help stratify tumors and gauge their sensitivity to stress as well as their suitability for SOCE inhibitor treatments. Furthermore, these tumors may be more sensitive to alternative chemotherapies which trigger apoptosis via ER stress by restoring the cellular sensitivity to ER stress through SOCE inhibitor treatment. Taken together, SOCE inhibition may pose an alternative treatment option for pancreatic cancer patients presenting increased STIM1 expression and a priori or acquired gemcitabine resistance.

## 5. Conclusions

Taken together, certain cellular features, such as genetic aberrations, are selected for upon external or internal cues. These features shape the cellular response to stimuli, promoting a transcriptional program which enables the cells to thrive under such adverse conditions. One such example, described and discussed extensively in this review is that of the genetic alteration of calcium regulatory genes. Calcium is a key second messenger that fine tunes the cellular response to various internal and external cues, ultimately shaping the cellular transcriptome upon stimuli. Thus, in order to identify changes in cellular transcriptional program dependencies, the characterization of genomic aberration events and their consequences in calcium and overall cellular signaling response is of utmost importance. This may aid in the identification of novel druggable targets in precision oncology.
